# Neck stiffness and bone osteolytic lesion in a 3-years old child: a case report

**DOI:** 10.1186/s13052-023-01534-4

**Published:** 2023-09-29

**Authors:** Carolina Carraro, Marco Rabusin, Flora Maria Murru, Lydie Ammar, Egidio Barbi, Alessandro Amaddeo, Giorgio Cozzi

**Affiliations:** 1 University of Trieste, Piazzale Europa, 1, 34127 Trieste, Italy; 2 Institute for Maternal and Child Health - IRCCS “Burlo Garofolo”, Trieste, Italy

**Keywords:** Torticollis, Neuroblastoma, Osteolytic lesion, Metastases, Case report

## Abstract

**Background:**

Neuroblastoma is the most frequent extracranial solid tumor occurring in childhood, representing approximately 28% of all cancers diagnosed in infants. Signs and symptoms of neuroblastoma vary with the site of development of the tumor and can mimic other diseases due to its extreme clinical variability. However, torticollis is not reported in the medical literature as a leading symptom of neuroblastoma.

**Case presentation:**

Here we report the case of a 3 years-old girl with fever and neck stiffness. Blood tests revealed a mild anemia and a rise in inflammatory markers. CT-scan showed a solid, heterogeneous, predominantly hypodense surrenal mass with eccentric calcification and extensive inhomogeneity of the vertebral metamers. Blood tests revealed raised serum levels of Neuron-Specific Enolase. At the 24-hours urine collection urinary catecholamines were greatly increased. A course of chemotherapy for neuroblastoma was promptly started with immediate clinical improvement.

**Conclusions:**

This case shows that the presence of torticollis could be a chief complaint of neuroblastoma. To our knowledge, neuroblastoma is not mentioned among life-threatening underlying conditions of torticollis in most recent literature reviews.

**Supplementary Information:**

The online version contains supplementary material available at 10.1186/s13052-023-01534-4.

## Background

Neuroblastoma is almost exclusively a disease of children. It represents approximately 28% of all cancers diagnosed in infants, being the third most common type of childhood cancer, after leukemia and brain tumors, and representing the most common solid extracranial tumor diagnosed by five years of age. Neuroblastoma is a malignant embryonal tumor of the neural crest cells, with a peak incidence in the first year of life and a slight prevalence in boys and Caucasians [1]. Although the majority of neuroblastomas are sporadic, 1–2% of them are familial. Inherited cases usually occur at an earlier age than sporadic cases (mean age 9 months), and a large proportion of them have bilateral adrenal or multifocal disease [2].

Neuroblastomas are heterogeneous tumors, varying in terms of location, clinical features, histopathologic appearance, and biological characteristics. They can arise anywhere throughout the sympathetic nervous system, although the most frequent site is the adrenal medulla (40%), followed by the paraspinal sympathetic ganglia. Its presentation can mimic other diseases, leading to diagnostic delay and misdiagnosis [3].

## Case presentation

A 3-year-old girl showed up to the Emergency Department (ED) because of fever and neck stiffness started 2 days before. Her medical history was remarkable for the presence of a recurrent mild right hip pain and limping started three weeks before, apparently after an upper airway infection with no significant improvement after the administration of anti-inflammatory therapy.

On admission, she was alert, with normal respiratory rate, febrile, pale and showed an evident neck stiffness. At physical examination, she complained of severe pain on neck movements in every direction. Movements of extra rotation of the right hip were also moderately limited. The abdominal examination was unremarkable.

Blood tests showed a mild anemia and a rise in inflammatory markers: WBC 11,460/mmc, HGB 9 g/dl, PLTs 333,000/mmc, CRP 98 mg/L (normal values: < 5.0 mg/L), ESR 120 mm/h.

A peripheral blood smear and an immunophenotype resulted unremarkable.

The hip ultrasound showed the presence of a mild joint effusion and synovial thickening. To rule out the presence of an abdominal mass, an abdominal ultrasound was performed, showing nothing remarkable.

The cervical spine X-ray was negative, whereas the pelvic X-ray showed an osteolytic lesion surrounded by a sclerotic rim near the right acetabulum and a cortical rarefaction of the left femoral neck. No lesions of the spine were detected.

Due to the presence of bone pain in multiple sites associated with osteolytic radiological findings, solid tumor metastasis and a chronic recurrent multifocal osteomyelitis (CRMO) were suspected. Thus, a total body magnetic resonance (MRI) was scheduled. Empirical intravenous antibiotics (oxacillin plus ceftriaxone) were started on the weak suspicion of an osteomyelitis.

Follow-up blood tests, however, revealed raised serum levels of Neuron-Specific Enolase (NSE) (66,5mcg/L, normal range 0-18mcg/L) and a slight increasement of LDH (353U/L, normal range 200-333U/L).

Therefore, a neck and abdominal CT-scan was performed instead of the scheduled MRI.

**Fig. 1:**
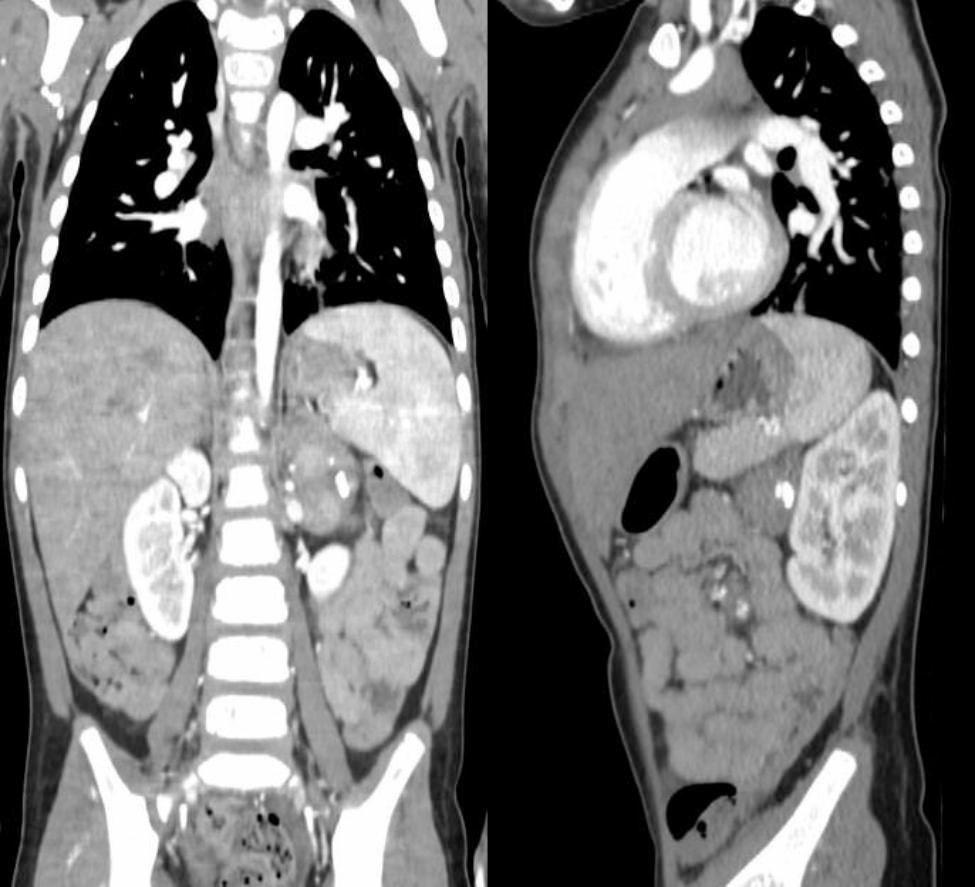
CT scan shows a solid, heterogeneous predominantly hypodense surrenal mass with eccentric calcification of dimensions of 2.5 cm x 2.7 cm x 4.5 cm

**Fig. 2:**
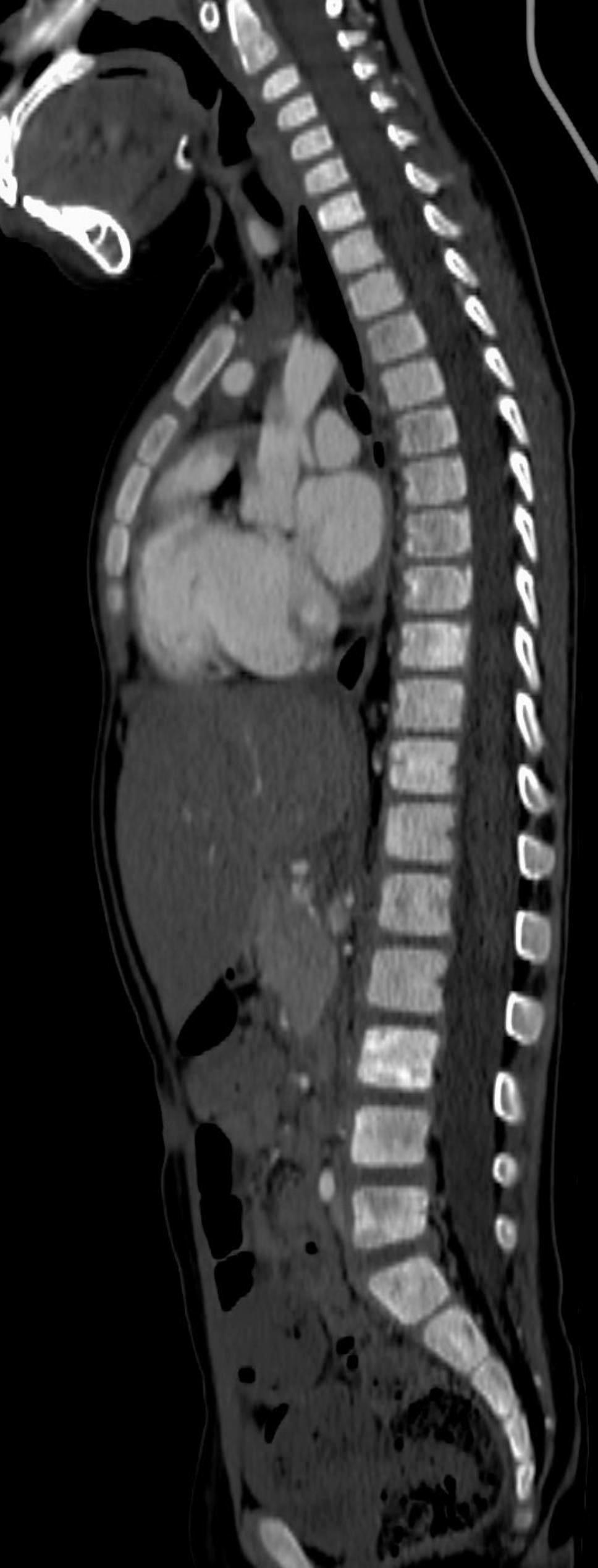
CT scan shows extensive inhomogeneity of the vertebral metamers with hyperdense and osteolytic areas

The CT-scan showed a solid, heterogeneous, predominantly hypodense surrenal mass with eccentric calcification of dimensions of 2.5 cm x 2.7 cm x 4.5 cm (Fig. 1). Imagines of the spine were suggestive of infiltration of the bone, showing extensive inhomogeneity of the vertebral metamers (Fig. 2). These features were considered indicators of neuroblastoma.

At the 24-hours urine collection, vanillylmandelic acid (VMA) and homovanillic acid (HVA) were increased (VMA 207.3 mg/24 h, HVA 137.1 mg/24 h). To have a whole-body disease assessment, MIBG scintigraphy was performed and showed high left adrenal uptake and widespread skeletal involvement. To confirm the diagnosis, bone marrow aspiration and biopsies were also carried out. The bone marrow aspiration revealed the presence of neoplastic infiltration > 50%, also confirmed by histological examination of the bone marrow biopsies. Amplification of the oncogene N-MYC emerged. The diagnosis of stage 4 neuroblastoma was then formalized.

The patient started the Rapid COJEC induction chemotherapy for high-risk neuroblastoma. In 24-hours the bone pain disappeared, the child was afebrile and the inflammatory markers eventually dropped significantly. In the last 9 months the girl completed the Rapid COJEC protocol. A CT-scan and a MIBG scintigraphy were carried out after the end of the chemotherapy, showing a complete remission of the skeletal disease. Furthermore, she underwent primary tumor resection and 2 autologous stem cell transplants. At the current state, her clinical conditions are stable.

## Discussion and conclusions

Signs and symptoms of neuroblastoma vary with the site of development of the tumor and can mimic other diseases due to its extreme clinical variability, with an increase in diagnostic complexity. Therefore, there is a high risk of diagnostic delay and misdiagnosis. Neuroblastomas can be initially asymptomatic and can be discovered as an incidental radiological finding especially in infants, or can cause symptoms from catecholamine secretion, mass effect or metastatic spread [3, 4]. Sign and symptoms of abdominal tumors, which represent approximately two thirds of primary neuroblastomas, may include abdominal mass, abdominal pain or fullness, constipation and urinary retention [5]. Moreover, large tumors can cause a compression of the venous and lymphatic drainage and of the renal artery, leading to hypertension. Neuroblastoma metastasizes by both lymphatic and hematogenous routes and almost 40% of neuroblastomas are metastatic at diagnosis. Hematogenous spread extends most often to bone, causing bone pain especially during ambulation, bone marrow, leading to cytopenia, skin, and liver, rarely to lung and brain parenchyma [6].

Musculoskeletal symptoms may occur in up to 25% of pediatric cancers and joint involvement may be present in 16% of cases. Limping is the most common manifestation, but other bones may be involved, causing different medical complaints, such as limb pain or back pain. In fact, neuroblastoma affects bones in 21% of cases [3, 7]. Lytic bone lesions have been described in metastatic neuroblastoma due to the activation of osteoclasts. Metastatic involvement of the bone marrow must be distinguished from lymphoma and leukemia.

Commonly, musculoskeletal symptoms and limping may be the presenting symptoms of rheumatic and infective diseases. This may lead to diagnostic delay, disease progression and a higher rate of treatment failure.

Factors that can help to discriminate malignancies from rheumatic disease are: limb and back bone pain, night pain, refusal to walk, arthropathy of the hip and the cervical spine, pain disproportionate to physical findings, and a discrepancy between inflammation signs (fever, arthritis) and the expected increase of blood cells count with relatively low WBC (in the range of 5000 mmc) and platelets (in the range of 100,000 mmc) [8].

Neuroblastoma is diagnosed on the basis of histological examination, combined with chemical profiling and imaging findings [9]. Ultrasounds are usually the first step to evaluate a child with a suspected abdominal mass, but a CT scan or an MRI are necessary to characterize the primary tumor and localize regional and distant metastases. In fact, even if the estimated overall diagnostic accuracy of US in detecting a pathological abdominal mass is high, the diagnostic accuracy in specific conditions such as neuroblastomas is relatively low (57,1%) [10]. Thus, US in a patient with adrenal neoplasm is a guide to further diagnostic workup. On the other hand, a negative abdominal US is not sufficient to exclude abdominal neuroblastomas. Blood tests could show cytopenia, due to bone marrow infiltration. Raised serum levels of Neuron-Specific Enolase have been found in all stages of neuroblastoma, although the incidence of increased concentration is greater in widespread and metastatic disease [11]. More than 90% of individuals with neuroblastoma also present elevated levels of VMA and HVA at the 24-hours urine collection and in single spot urines. To have a whole-body disease assessment, I-123 metaiodobenzylguanidine (mIBG) scintigraphy and bone marrow aspiration should also be performed.

Children with neuroblastoma could have widely different outcomes. In fact, neuroblastomas are remarkable for their broad spectrum of clinical behavior, which can range from spontaneous regression to aggressive disease with metastatic dissemination. According to the International Neuroblastoma Risk Group (INRG) staging system, patients can be partitioned into 4 risk groups for treatment. Patients with low- and intermediate-risk disease are variably treated with surgical resection or moderate doses of chemotherapy followed by surgical resection. Infants with stage 4 S/MS neuroblastoma may undergo spontaneous regression. Asyntomatic infants with stage 4 S neuroblastoma can be safely observed and followed with a strict clinical and radiological follow-up [12]. Current standard treatment for high-risk patients includes 4 treatment blocks: induction with chemotherapy, surgery on primary site of disease, consolidation involving the administration of high dose chemotherapy combined with autologous stem cell transplantation and radiotherapy, and maintenance with immunotherapy and 11 cis-retinol [13].

This case shows that the presence of torticollis could be a chief complaint of neuroblastoma. In a child with an unexplainable torticollis, we should always consider bone involvement by a metastatic neuroblastoma.

To our knowledge, neuroblastoma is not mentioned among life-threatening underlying conditions of torticollis in most recent literature reviews [14]. In fact, while posterior fossa tumors are always suspected in front of torticollis in infants, torticollis is not usually considered as an alarming sign of possible neuroblastoma. In a cohort study in which 392 pediatric patients with torticollis were enrolled, none had neuroblastoma [15].

This case reminds general practitioners that in presence of unexplained torticollis in a toddler a neuroblastoma should be ruled out. Pediatricians play a crucial role in the diagnosis of neuroblastoma and should be aware of the multitude of clinical manifestations of this elusive tumor [3].

### Electronic supplementary material

Below is the link to the electronic supplementary material.

Supplementary Material 1

## Data Availability

Not applicable.

## List of abbreviations

ED: Emergency department
WBC: White blood cells
HGB: Hemoglobin
PLTs: Platelets
CRP: C-reactive protein
ESR: Erythrocite sedimentation rate
CRMO: Chronic multifocal recurrent osteomyelitis
LDH: Lactate dehydrogenase
MRI: Magnetic resonance imaging
CT: Computed tomography
NSE: Neuron specific enolase
VMA: Vanillylmandelic acid
HVA: Homovanillic acid
mIBG: I-metaiodobenzylguanidine
INRG: International neuroblastoma risk group
