# Supplementary material for: Neck stiffness and bone osteolytic lesion in a 3-years old child: a case report
Source: Ital J Pediatr. 2023 Sep 29;49:132. doi: 10.1186/s13052-023-01534-4 (PMC10543269; doi:10.1186/s13052-023-01534-4)
Supplement: Supplementary file 1 — Supplementary Material 1 [file 13052_2023_1534_MOESM1_ESM.docx]

Dear Editor,

Thank you very much for having been able to provide feedback for our paper ITJP-D-23-00415 entitled "Neck stiffness and bone osteolytic lesion in infants: a case report”. Please find below the replies to the issues raised by the Reviewers.

We changed the manuscript following Your suggestions. See text

**Reviewer #1**:
-**Request 1**, PP 3, line 29: *I think some other details about NB were known (NSE, AVM?) before starting chemotherapy: can you briefly add them?*

**Authors:** we added details about NB as you asked (lines 29-31)

-**Request 2**, P4 line 37: *I suggest to substitute child to toddler; torcicollis can be onset symptom of older children too (personal observation).*

**Authors:** we substituted child to toddler as you suggested (line 38)

-**Request 3**, PP 4, line 4: *please report CRP normal value.*

**Authors:** we added CRP normal values (<5.0 mg/L) (line 68)

-**Request 4**, PP4, lines 75-76: *"Empirical intravenous antibiotics (oxacillin plus ceftriaxone) were started on the weak suspicion of an osteomyelitis". I suggest to put this therapy attempt description after "osteomyelitis (CRMO) were suspected" (line 79).*

**Authors**: we put the sentence *"Empirical intravenous antibiotics (oxacillin plus ceftriaxone) were started on the weak suspicion of an osteomyelitis"* at line 82, after *"osteomyelitis (CRMO) were suspected".*

-**Request 5**, PP 5, line 104*: please add "especially in infants" about incidental radiological finding.*

**Authors:** we added “especially in infants” as you suggested (line 115)

-**Request 6**, PP 6, *line 106-119: please add some references.*

**Authors**: we added some references (lines 111-125)

-**Request 7**, PP 6, line 115: "*Metastatic involvement of the liver is common in infants and can cause respiratory compromise": this sentence it's a bit confounding and can be abrogate.*

**Authors:** we abrogated the sentence as you suggested.

-**Request 8**, PP 6, line 119-121: *"almost as much as leukemia, which is the tumor that most frequently involves the bone structure (excluding primary bone cancers)": this sentence can be abrogate.*

**Authors:** we abrogated the sentence as you suggested.

-**Request 9**: you suggested to modify the final part of this paragraph (p 6, lines 116-122), adding something about the clinical presentation of other cancers and reporting about risks of delayed diagnosis or misdiagnosis.

**Authors:** we related possible onset symptoms of NB to those of other malignancies. For example, cytopenia may indicate a metastatic NB or could suggest a leukemia. We also explained the consequences of misdiagnosis: *“This may lead to diagnostic delay, disease progression and a higher rate of treatment failure”* (lines 127-138).

-**Request 10**, P 7, line 39. *I suggest to substitute "can also" with "should also".*

**Authors:** we substituted *"can also" with "should also"* as you recommended (line 161).

-**Request 11**, P 7, line 148: *after regression should be add something about the need of a strict radiological FUP.*

**Authors:** we added a paragraph to explain the importance of a close clinical and radiological FUP in asyntomatic stage 4S NB (lines 169-171).

-**Request 12**, PP 7 156-57: *"In fact, osteolytic lesions may be indicator of metastatic neuroblastoma. "This sentence is pleonastic and can be omitted.*

**Authors:** we abrogated the sentence.


**Reviewer #2**:

-**Reviewer’s requests on case presentation:** you asked to describe the abdominal physical examination. In addition, you also requested a comment on the abdominal US, which did not identify the mass and on MRI findings. Furthermore, you recommended to add the dimensions of the mass on CT scans and to add details about the follow-up.

**Authors:** the abdominal physical examination was unremarkable. On palpation of the abdomen pain was not reported and no organomegaly or masses were appreciated. Regarding the abdomen US, we added an explanatory paragraph in the discussion section (lines 149-154). We are aware that MRI would have been the best radiological examination to perform. However, we performed a CT scan, which revealed an abdominal mass of dimensions of 2.5x2.7x4.5 cm. Finally, we added details about the follow-up (lines 104-109).

-**Reviewer’s requests on discussion:** you suggested to change the position of the sentence "Metastatic involvement of the liver is common in infants and can cause respiratory compromise" and to add references in the discussion (lines 102 - 117). Finally, you suggested to anticipate the sentence "Therefore, there is a high risk of diagnostic delay and misdiagnosis" in the first part of the discussion.

**Authors:** we abrogated the sentence "Metastatic involvement of the liver is common in infants and can cause respiratory compromise", as it was a bit confounding and changed the position of the sentence "Therefore, there is a high risk of diagnostic delay and misdiagnosis" (lines 113-114). Moreover, we added references as you suggested to the discussion section (lines 111-125).

Finally, although it was not among your requests/suggestions, we have agreed to change, if possible, the title of the manuscript.

From: *Neck stiffness and bone osteolytic lesion in infants: a case report*

To: *Neck stiffness and bone osteolytic lesion in a 3-years old child: a case report.*

We think that the new title is more pertinent to the subject of the case report.

We hope that our manuscript may now be acceptable for publication in the Journal. We look forward hearing from you at your earliest convenience.

Yours sincerely,

Carolina Carraro
